# Cardiac arrhythmia detection using deep learning approach and time frequency representation of ECG signals

**DOI:** 10.1186/s12911-023-02326-w

**Published:** 2023-10-19

**Authors:** Yared Daniel Daydulo, Bheema Lingaiah Thamineni, Ahmed Ali Dawud

**Affiliations:** 1 Department of Biomedical Engineering, Dilla University Referral Hospital, Dilla, Ethiopia; 2 School of Biomedical Engineering, Jimma Institute of Technology, Jimma University, Jimma, Ethiopia

**Keywords:** AlexNet, CVD, ECG, Deep learning, Morse wavelet, ResNet50

## Abstract

**Background:**

Cardiac arrhythmia is a cardiovascular disorder characterized by disturbances in the heartbeat caused by electrical conduction anomalies in cardiac muscle. Clinically, ECG machines are utilized to diagnose and monitor cardiac arrhythmia noninvasively. Since ECG signals are dynamic in nature and depict various complex information, visual assessment and analysis are time consuming and very difficult. Therefore, an automated system that can assist physicians in the easy detection of arrhythmia is needed.

**Method:**

The main objective of this study was to create an automated deep learning model capable of accurately classifying ECG signals into three categories: cardiac arrhythmia (ARR), congestive heart failure (CHF), and normal sinus rhythm (NSR). To achieve this, ECG data from the MIT-BIH and BIDMC databases available on PhysioNet were preprocessed and segmented before being utilized for deep learning model training. Pretrained models, ResNet 50 and AlexNet, were fine-tuned and configured to achieve optimal classification results. The main outcome measures for evaluating the performance of the model were F-measure, recall, precision, sensitivity, specificity, and accuracy, obtained from a multi-class confusion matrix.

**Result:**

The proposed deep learning model showed overall classification accuracy of 99.2%, average sensitivity of 99.2%, average specificity of 99.6%, average recall, precision and F- measure of 99.2% of test data.

**Conclusion:**

The proposed work introduced a robust approach for the classification of arrhythmias in comparison with the most recent state of the art and will reduce the diagnosis time and error that occurs in the visual investigation of ECG signals.

## Background

The cardiovascular system is one of the physiological systems of the human body that is characterized by a complex interaction of several organs and tissues including the heart and blood vessels [1]. According to a WHO report, CVD are identified as the leading cause of human death in the world [2]. It is estimated that more than 17 million people are known to die yearly by CVDs. Moreover, 75% of the total CVD deaths occur in middle and low-income countries [3].

**Fig. 1:**
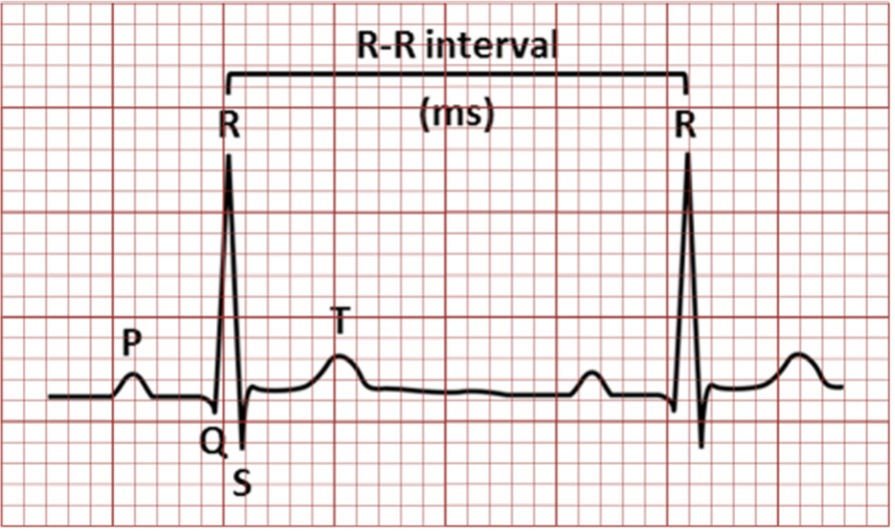
ECG waveform

The number of deaths due to CVD is expected to rise up to 23 million by the year 2030. Beside this, treatment of CVD, including cost of diagnosis and medication, is very expensive. In low- and middle-income countries, the cost of treatment is estimated to be approximately 3.8 trillion dollars from 2011 to 2025 [4]. Of the deaths that occurred due to cardiovascular disorders, deaths due to cardiac arrhythmias were the most prominent. Cardiac arrhythmias are disturbances in the heartbeat due to electrical conduction anomalies in cardiac muscle [5]. Clinically, such anomalies are diagnosed using electrocardiograms, which are the gold standard diagnostic and monitoring tool in modern medicine. ECG records bioelectric potential generated due to electrical activity of the hearts by using non-invasive skin electrodes attached on the chest. Hence, the recorded information about condition of heart is represented in amplitude and duration in the form of waves named P-QRS-T as shown in Fig. 1 below. This waveform contains significant information about the condition of heart and nature of disorders afflicting it. ECG based monitoring in the clinical care setting ranges from reading the basic rhythm of the heart to diagnose of complex disorders such as arrhythmias. Any abnormality of heart rhythm or changes in patterns of P-QRS-T wave is a manifestation of cardiac arrhythmia that could be identified by assessment of the recorded signal.

Physicians use Electrocardiography techniques for diagnoses and assessment of cardiovascular diseases. In this technique ECG signal is visually assessed visually by experts. Due to the non-stationary nature of ECG signals, anomalies may not occur at all times of recording. Hence, correct diagnosis of cardiac disorder from the ECG signal requires observation and analysis of the recording for long period of times. However, examining of ECG for a long hour requires large amounts of data and this turns out to be tiresome and time consuming. Moreover, due to the amount of data used in analysis, the probability of missing data is very high. Therefore, an automated system that distinguishes abnormal and normal ECG signals is required to assist doctors for easy identification of cardiac arrhythmia accurately.

Several researchers developed diverse, intelligent systems for the analysis of ECG signals automatically. As ECG is a one-dimensional (1-D) signal representing a time series, a machine learning approach-based intelligent system is one of the methods applied in the classification of ECG signals. It mainly relies on extracting significant features and selecting the best set of features used for classification of heartbeats. The study aimed to classify heartbeats into two classes based on their morphology using a machine learning approach was performed by Alfaras et al. [6]. They used the MIT-BIH ARR and AAMI ECG databases and implemented a classification model named ensemble of echo state networks. Conventional ECG signal preprocessing, feature extraction, and feature selection techniques were implemented in the study. Thus, positive predictive value of 86.1% is achieved for ventricular ectopic beats using the single lead II where as a positive predictive value of 75.1% is achieved while using lead V1. Saira et al. [7] proposed a model for heartbeat classification using fractional- Fourier-transform algorithm-based feature extraction and the MLP classifier. After extracting various features from the time series ECG, four sets of features were selected for final classification. In evaluating the model performance using the MIT-BIH database, an overall accuracy of 80% was achieved for classification of CVD, while evaluating in the case of the Shaoxing People’s Hospital database, an accuracy of 90.7% was achieved. Another machine learning approach for the classification of ECG signals was proposed by Kishore et al. [8]. The genetic algorithm is applied for feature extraction, and the radial basis function neural network is implemented for classification. In the overall classification of heartbeats into six classes, the proposed RBFNN achieved 98.5% accuracy.

In recent years, the development of deep learning models has created a breakthrough in addressing several problems that exist with traditional machine learning approaches. It mainly differs from traditional machine learning, as it does not require a handcrafted feature extraction and selection strategy by automatically learning significant information from data. Recently, several studies have been carried out using deep learning-based ECG signal classification and have shown promising performance.

Byeon et al. [9] Proposed a customized deep learning model named EECGNet for biometric classification of ECG signals using the Physikalisch-Technische Bundesanstalt (PTB) database. They used a 2D image of the ECG signal as input for the model and achieved a classification accuracy of 98.89%. Although, the study focused on the application of ECG signals for biometric purposes, the results suggest that a deep learning approach could provide promising results if implemented in arrhythmia classification. Another study aimed at detection and classification of fall using ECG signals and deep transfer learning approach was carried out in [10]. They used for the time frequency representation of ECG signals to convert 1D ECG signals into 2D signals and reported accuracy of 97.36% using a pretrained AlexNet model. Another study that was aimed at classifying the ECG signal QRS complex into three classes using global average based 2-D CNN modelling was conducted in [11]. The study is carried out by using a publicly available European DT-T database and achieved a promising classification accuracy of 99.23%.

Atrial fibrillation detection from ECG signals using a pretrained EfficientNet B0 convolution neural network model was proposed in [12]. As the model requires a 2D input image, they used STFT to generate 2D image of ECG signals. A classification accuracy of 97.3% was achieved when testing the model using the PhysioNet Computing in Cardiology Challenge dataset. A hybrid deep learning model was used to classify the PhysioNet MIT-BIH arrhythmia database and reported accuracy of 97.15% [13]. Karthiga et al. proposed a deep learning CNN for classification of ECG signals using MIT-BIH arrhythmia data and 91.92% classification accuracy was reported [14]. A hybrid deep learning model named CNN—LSTM was proposed in [15] for cardiac arrhythmia detection from CWT images of ECG recordings. According to reported result, the proposed approach provided 98.0%, 96.0%, and 98.0% accuracy for ARR, CHF, and NSR, respectively.

Despite the good performance achieved by the traditional machine learning approach in [5–7], the dependence of the model on handy feature extraction and selection affects the performance of the model. One of the problems with traditional machine learning approaches is the handcrafted feature extraction and selection approach. Thus, one may select few features such as morphological feature [6], R-R interval and transitory features [7] that address the problem at hand, which may lead to loss of significant information, whereas others may select many features that are not valuable for the problem at hand, which leads to the computational complexity of a model. However, deep learning differs from feature extraction-based machine learning because it has the capability of learning from data automatically. Hence, there is no need for the extraction and selection of features, as the model performs these steps automatically based on the fine-tuned hyperparameters. Currently, researchers on ECG signals are inspired by the art of deep learning, and promising classification performance has been reported in the literature [8–15]. Although the literatures[8, 9] implemented a deep learning model for biometric and fall detection, their positive motivational methodologies are considered in the present work.

The primary objective of this study is to enhance the field of cardiac arrhythmia detection of ECG signals by utilizing a Morse-based time–frequency representation and transfer learning methodology. The study's key contributions can be summarized as follows: providing an overview of various machine learning and deep learning approaches for detecting cardiovascular diseases from ECG signals; Discussing the importance of using an analytic Morse wavelet to transform time series signals into 2D images of time–frequency representation; Describing the architecture, components, and parameters of proposed transfer learning models used in optimization; Fine-tuning and optimizing pre-trained AlexNet and ReNet 50 to significantly improve classification performance; and presenting detailed results of training, validation, and testing of the proposed models, comparing them with related state-of-the-art approaches.

## Methods

### Data source

**Fig. 2:**
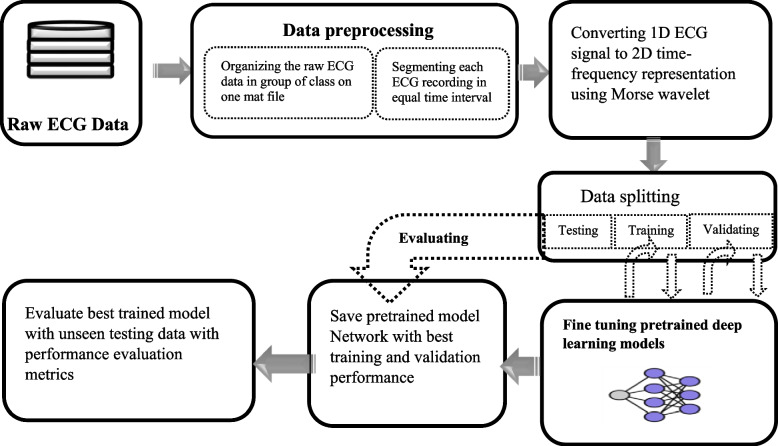
Schemes of overall methodology

In the present study, three datasets from the Physionet database were used to train models and to evaluate their performance. The database is available online in a Physionet official link (https://archive.physionet.org/physiobank/database, which is accessed on 1 March 2023). The first data are obtained from the Physionet sub database named the MIT–BIH arrhythmias database and contain 96 ECG recordings of participants with congestive heart failure who are aged within the range of 34 to 79. All ECG recordings of the MIT-BIH cardiac arrhythmia database were digitized at 128 samples per second [16, 17]. The second database contains 36 ECG recordings which are class of normal sinus rhythm obtained from the Physionet sub database named the MIT-BIH normal sinus rhythm database of the Physionet database accessed through the official link (https://www.physionet.org/physiobank/database/nsrdb/). The age of patients in this database comprised 18 subjects from 26 to 45 years and thirteen subjects aged from 20 to 50 years old. All of normal sinus rhythm class ECG recording is sampled at sampling frequency of 128 Hz, so that each sample point is at intervals of 0.00781 s [17]. The third database contains 30 ECG recordings taken from the BIDMC congestive heart failure database accessed through the link (https://www.physionet.org/physiobank/database/chfdb/), and this database comprises long-term recordings of 15 participants with severe congestive heart failure and aged between 22 to 71 years [16, 18]. Figure 2 explain overall methodology.

### ECG data preprocessing

**Fig. 3:**
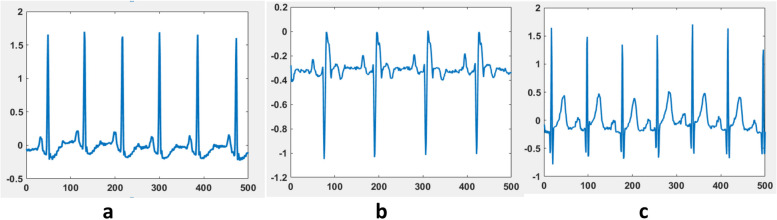
Segmented ECG recording of type ARR (a), CHF (b) and NSR (c)

Organizing the ECG data obtained from three different sources of the PhysioNet database and segmenting each class of recording are the key parts of the ECG data preprocessing procedure conducted in this study. The data used in this study were collected from 162 subjects, of whom 96 recordings were for arrhythmia, 30 recordings were for congestive heart failure, and the remaining 36 recordings accounted for the normal sinus rhythm. Each patient ECG recording consisted of 65,536 samples. Since sending this much length of ECG signal as one data affects the performance of the deep learning model due to degradation, segmenting each recording to equal length of the segments is needed. Thus, each recording that consisted of 65536 samples was segmented into 20 pieces, and each piece consisted of 500 samples. Moreover, to make the distribution of data equal to each class, only 30 patient ECG recordings per class were chosen. In total, 1200 data points were generated for each class in this preprocessing stage. Figure 3 below shows segmented ECG recording for each class of recording.

### Conversion of 1D time series ECG signal to 2D image using morse wavelet

CNN model requires input data in the form of images. However, most biomedical signals come in the form of 1D time series signals. Several researchers have used various techniques to convert one-dimensional time series signals to 2D images. The study conducted by Wasimuddin et al. [11] implemented morphological shape images of ECG signals to feed a CNN network. Another study conducted by Wei et al. [19] implemented a short-time Fourier transform to convert a 1D ECG signal to an image as a spectrogram. Recently, several researchers have been inspired by the time frequency representation of time series biomedical signals using continuous wavelet transforms in the form of scalograms. From the continuous wavelet family, the analytic wavelet family is widely implemented for the 2D representation of ECG signals [20]. Electromyograms (EMGs) [21] and, very recently, Daydulo et al. [22] implemented family of analytic wavelets named generalized Morse wavelets to convert the 1d FHR signal of cardiotocogram (CTG) recordings to scalogram images. Generalized Morse wavelet is a family of analytic wavelet which is well known by its excellent properties of being exactly analytic wavelet. Moreover, it has no support for negative frequency [22, 23]. Using the generalized Morse wavelet for time frequency representation of nonstationary signals such as ECG is an ideal choice as it determines short duration, frequency, localize discontinuities, amplitude, transient and joint time–frequency representation of time-varying amplitude [24]. Furthermore, Morse parameters flexibility makes it versatile to be adapted to all other analytic wavelet family classes [23].

Unlike other analytic wavelet with one parameter, Morse has two important parameters that makes it versatile and adoptable in comparison with other AW. For Morse wavelet written in form of ${\varphi }_{P,\gamma }(t)$, it’s equation in frequency domain can be defined as shown in Eq. 1 [25].

1 $${\varphi }_{P.\gamma }\left(\omega \right)=U\left(\omega \right){\alpha }_{P,\gamma }{\omega }^{\frac{{P}^{2}}{\gamma }}{e}^{-{\omega }^{\gamma }}$$

In the above equation P^2^ represents time bandwidth product of the wavelet, whereas $\gamma$ represents symmetry parameter, e is constant number commonly called Euler’s number and its approximate value is $2.71828$,$U(\omega )$ is the unit step function and ${\alpha }_{P,\gamma }$ is the normalizing constant. Time bandwidth parameter of Morse wavelet is also defined with the formula ${P}^{2}= \gamma * \beta$ [24]. Hence, Eq. 1 will be modified as Eq. 2, which is written using the $\beta and \gamma$ parameter.

2 $${\varphi }_{\beta .\gamma }\left(\omega \right){\alpha }_{\beta ,\gamma }{\omega }^{\beta }{e}^{-{\omega }^{\gamma }}$$

By using the $\upgamma$ value equal to 3 the skewness of the Morse wavelet becomes 0, and due to this the wavelet exhibits minimum Heisenberg area still being exactly analytic wavelet [14]. Beside this, at this value of $\gamma$, the wavelet becomes the most symmetric and the most Gaussian wavelet still having a very low Heisenberg area [26]. Therefore, in this work the $\gamma$ value of 3 is assigned to represent the ECG signal in time and frequency image.

**Fig. 4:**
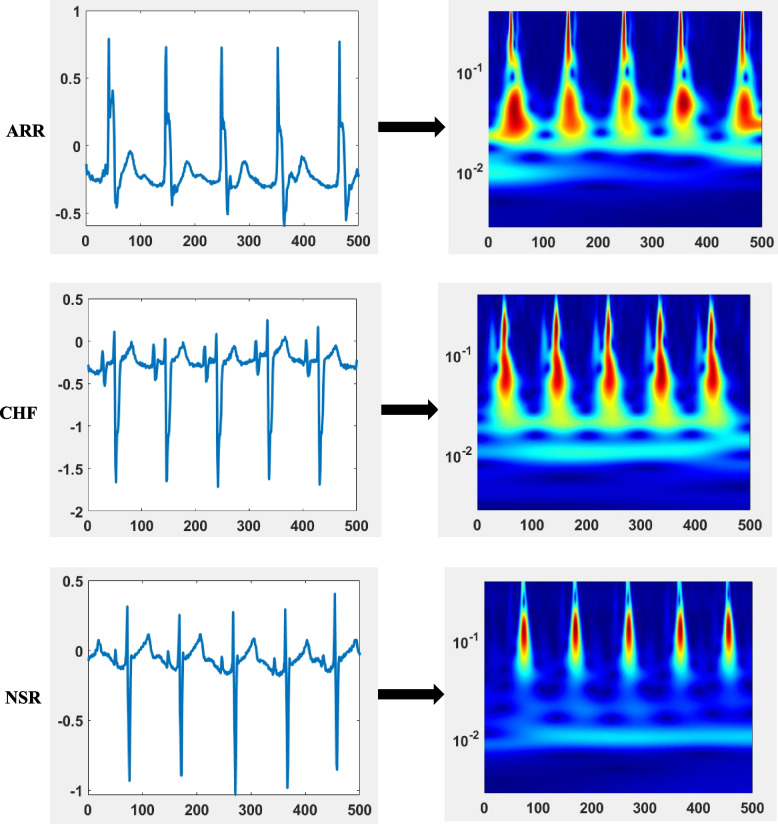
Conversion of time series ECG recording of type of ARR, CHF and NSR to image

The P^2^ parameter of the Morse wavelet determines how many oscillations fit in the envelope [25]. All the parameters of Morse wavelet are analysed using MATLAB ‘cwtfilterbank’ for time frequency representation of ECG. The MATLAB ‘cwtfilterbank’ design was done considering the Morse wavelet and ECG signal parameters. Thus, signal length of 500 samples, frequency of 128 Hz, default value of P^2^ which is 60 and voice for an octave value of 12 are implemented for current work. Figure 4 shows the conversion of a 1D ECG signal to a 2D image using the generalized Morse wavelet with the aforementioned parameter value for each class of recording.

### The concept of transfer learning in deep learning

Transferring the weight value of pretrained network that has trained on a huge dataset to another new neural network is called transfer learning. In this method developing new models and training all of its upper and deeper networks is avoided [27], Therefore, the speed of training and updating networks of the model is significantly quicker than developing new models and training all of its networks from scratch. This technique is preferable when there is a limitation in amount dataset and computational power. Hence, using pre-trained networks provides quite competitive results against training from scratch. Pre trained models such as; ResNet, AlexNet, GoogLeNet, NASA Net, etc. are deep learning models, well known by their performance and can be implemented for transfer learning [28]. It’s very essential to consider varying characteristics of the pretrained network while selecting them for a problem at hand. Considering the pretrained network accuracy, it’s size and speed of training are very crucial characteristic. In this work the major factor that affects the efficiency of the model are considered such as absence of the GPU, inadequate memory space and accuracy, so that AlexNet and ResNet50 models are selected.

#### AlexNet model

AlexNet model is the first deeper model in comparison with the models developed prior to it and obtained first place in competition of ImgeNet held on 2012 [29]. AlexNet’s superiority in classification over previous methods created a revolution in the field of machine learning; this created the point where aspiration in deep learning increased tremendously.

The introduction of new parameters such as the ReLU, LRN and dropout in AlexNet has attracted the attention of the deep learning community. Replacement of traditional sigmoid function by ReLU in AlexNet considerably enhances the training speed and avoid overfitting. Furthermore, other magnificent strategy in AleNet is LRN that promotes to promote convergence, and dropout is also introduced in AlexNet to prevent overfitting [30].

**Fig. 5:**
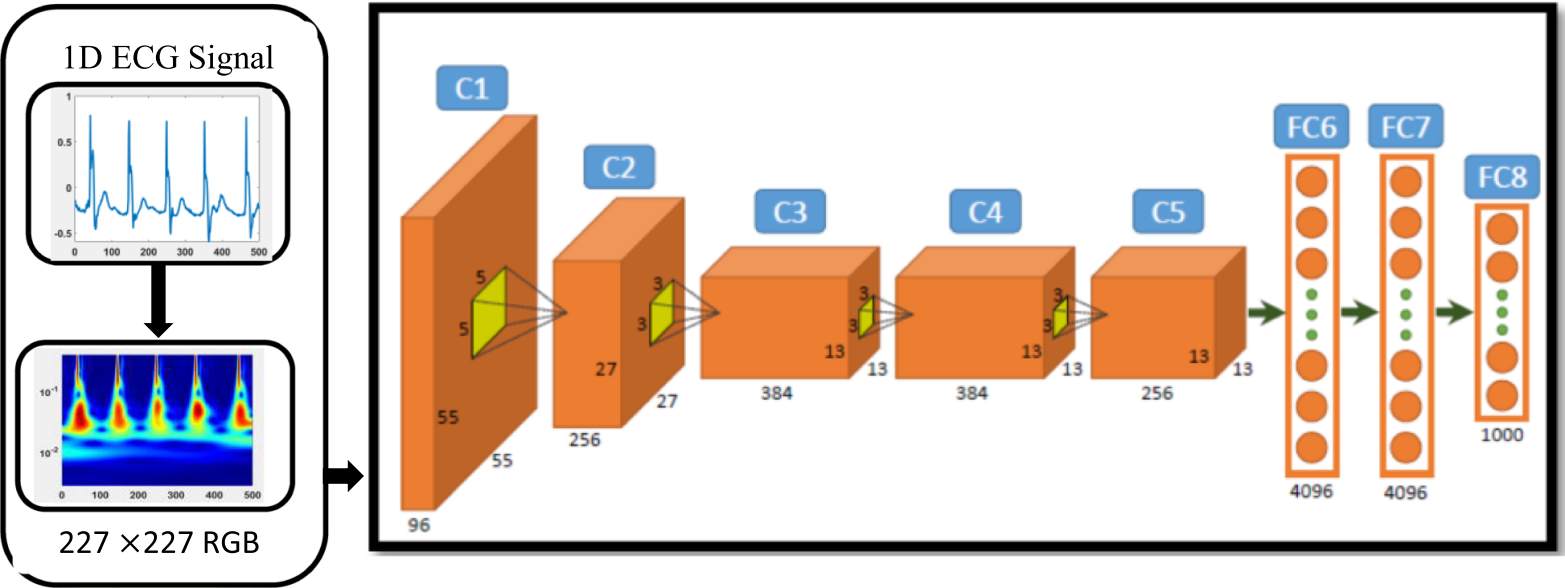
Overall architecture of AlexNet Model proposed in this work

As shown in Fig. 5 below, the input image for AlexNet must be an RGB image of 227 $\times$ 227; if not this size of image, the model will suffer from over fitting.

Moreover, the input image has to be changed to an RGB colour if it was not RGB, so a ‘jet color map’ was implemented for representing the signal by CWT in the current study. The first convolution layer represented by C1 in Fig. 5 carries out convolution and max-pooling with local response localization, in this layer 96 filters that are 11 by 11 stride size are used. Max pooling operation carry out by filters that has size of 5 by 5 and with stride size of 2. The max pooling operation is performed with 3 by 3 filters with a stride size of 2. In the second layer which is represented by C2, a similar operation is performed by filter sized 5 by 5. In the third, fourth and fifth convolutional layers, 384 and 256 feature maps are used with 3 by 3 filters. At the end of the network two fully connected layers are used with dropout followed by a softmax layer. Alex net has the following features that makes it suitable for analysis and classification tasks; deep structure in comparison to models developed prior to it, simple structure with very low memory requirement and fast training time [31].

#### ResNet 50 model

After AlexNet created a breakthrough in the field of machine learning, the deep learning researcher started to design a deeper network to achieve high accuracy. However, the problem of vanishing gradient effect is observed while training deeper network by cascading more layers.

**Fig. 6:**
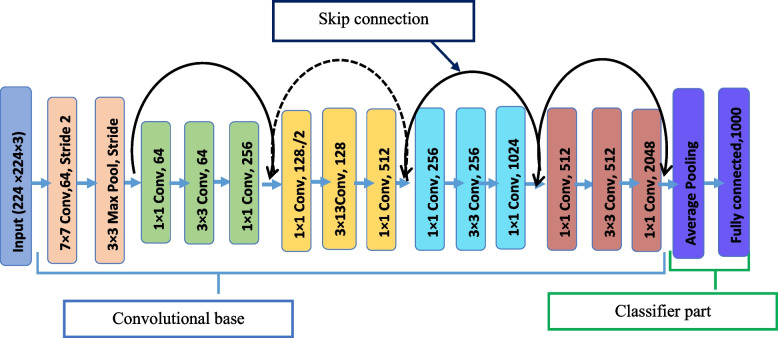
Architecture of the ResNet 50 model

Hence, deep network training will not converge, and accuracy will either start to degrade or saturate at a particular value. This problem is solved by introducing a residual neural network model. Residual network is a specific type of neural network introduced by 2015 and well known by its short name ResNet [31]. Comparing with all deep networks designed previous to it, ResNet is more in depth and it allows training of its upper and deeper networks effectively without hampering by vanishing gradient effect. The problem of vanishing gradient affects deep networks that were designed previous to ResNet as they didn’t have an identity shortcut or skip connection that helps the deeper network to reuse input features generated by upper layers and forward it to the next layer [32], as shown in Fig. 6. ResNet is one of the most powerful deep neural networks that achieved excellent performance on the ILSVRC 2015 image identification competition. In addition, ResNet won first place on ImageNet detection, ImageNet localization, COCO detection and COCO segmentation in ILSVRC and COCO 2015 competition [33]. Using ResNet as transfer learning approach is an ideal choice as it has some key features such as batch normalization, bottle neck design and identity connection. The batch normalization and bottle neck design in ResNet helps to increase performance of the model. Moreover, the identity connection helps to the deep networks to not be affected by vanishing gradient effect [31].

### Hyper parameters of pretrained models

Before training a deep neural network, the hyperparameters have to be set and adjusted in order to achieve reasonable results. Once they are set prior of training, they cannot be changed during training [34]. Thus, it’s very crucial to select the appropriate hyperparameters for better performance achievement of deep neural network. Some of the main hyperparameters set in this study are briefed below.

#### Size of min batch

A set of data points that are processed by the networks in one full iteration is referred as batch size. Size of the batch can be set from 1 to any appropriate number of sizes depending on GPU resources. It has a significant effect on training speed, loss convergence and number of iterations. In this work the batch size of 30 was utilized.

#### Epoch number

The epoch is a term used to refer the frequency that all training data are seen by the deep learning network. One epoch means one cycle of analysing all training data by the network. After the first epoch has completed, the next epoch will start as the second epoch. Thus, the maximum epochs of 15 were set in this work.

#### Learning rate

During training the weight of the network is adjusted several times with respect to loss of gradient and this adjustment of the network is determined by learning rate. If the learning rate is too small it takes much time to converge the loss, however the training loss becomes more stable. If it is higher, the loss will not be stable, but the speed of convergence becomes fast. So that selecting appropriate learning rate is mandatory. In the current study, the best performance was achieved for a learning rate value of 0.0001.

#### Optimizer

During training process, the value of neural network parameters such as weight, learning rate are adjusted in order to reduce loss and provide the best performance result as much as possible. These adjustment parameters are performed with the help of some set of rules named optimizers. Varies types of optimizers are available such as Adaptive gradient (AdaGrad), Stochastic Gradient Descent (SGD), adaptive moment estimation (ADAM) and Root mean squared propagation (RMSProp). In this study ADAM optimizer, which is replacement of SGD is used. Basically ADAM combines best features of RMSprop and AdaGrad [35] hence, it has the ability to easily obtain loss and fast convergence.

#### Activation function

Activation function performs functional mapping of the output neuron for the given input and weights. ReLU activation function is the most appropriate activation function that makes training of network fast [36]. In addition, it also helps to avoid the problem of vanishing gradient.

### Performance evaluation metrics

Training and testing performance of neural network has to be evaluated once it is built up and configured. Hence, the performance evaluation metrics named; recall (Re), precision (PR), sensitivity (Se), specificity (Sp) and accuracy (Ac) indicators are adopted from the confusion matrix in this work.

3 $$\mathrm{Accur}a\mathrm{cy}=\frac{\mathrm{Number}\;\mathrm{of}\;\mathrm{true}\;\mathrm{posetive}\;+\;\mathrm{Number}\;\mathrm{of}\;\mathrm{true}\;\mathrm{negative}}{\mathrm{Number}\;\mathrm{of}\;\mathrm{total}\;\mathrm{negative}\;+\;\mathrm{Number}\;\mathrm{of}\;\mathrm{total}\;\mathrm{positive}}$$

4 $$\mathrm{Sensitivity}=\frac{\mathrm{Number}\;\mathrm{of}\;\mathrm{true}\;\mathrm{posetive}}{\mathrm{Number}\;\mathrm{of}\;\mathrm{true}\;\mathrm{posetives}\;+\;\mathrm{Number}\;\mathrm{of}\;\mathrm{false}\;\mathrm{negatives}}$$

5 $$\mathrm{Recall}=\frac{\mathrm{Number}\;\mathrm{of}\;\mathrm{true}\;\mathrm{posetive}}{\mathrm{Number}\;\mathrm{of}\;\mathrm{true}\;\mathrm{posetives}\;+\;\mathrm{Number}\;\mathrm{of}\;\mathrm{false}\;\mathrm{negatives}}$$

6 $$\mathrm{Precision}=\frac{\mathrm{Number}\;\mathrm{of}\;\mathrm{true}\;\mathrm{posetive}}{\mathrm{Number}\;\mathrm{of}\;\mathrm{true}\;\mathrm{posetives}\;+\;\mathrm{Number}\;\mathrm{of}\;\mathrm{false}\;\mathrm{posetive}}$$

7 $$\mathrm{Specificity}=\frac{\mathrm{Number}\;\mathrm{of}\;\mathrm{true}\;\mathrm{Negative}}{\mathrm{Number}\;\mathrm{of}\;\mathrm{true}\;\mathrm{negatives}\;+\;\mathrm{Number}\;\mathrm{of}\;\mathrm{false}\;\mathrm{posetive}}$$

8 $$\mathrm F-\mathrm{measure}=2\ast\frac{\left(\mathrm{Precision}\ast\mathrm{Recall}\right)}{\mathrm{Precision}\;+\;\mathrm{Recall}}$$

### Data split scheme

**Fig. 7:**
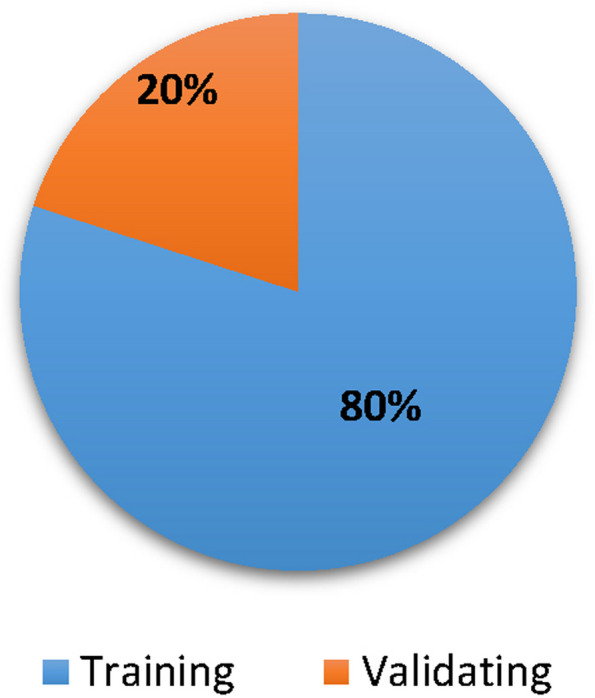
Data splitting scheme

The dataset split was performed on each class ECG signal to distribute the overall data into training and validation group. In order to do so a well-known 20% and 80% splitting scheme is used to categorize the data into validation and training respectively as depicted in Fig. 7. Therefore, from 1200 data points, 980 data points, which accounted for 80%, were used for training for each class. Then, the remaining 20%, which is 240 data points, was used for validation. The deep neural network trains with the training dataset and its performance during training is evaluated using validation dataset. In order to evaluate the generalizability of the model unseen test data set is used.

## Result

### Training result

After preprocessing and time frequency representation, 3600-time frequency images were generated, and each class accounted 1200 images. According to data splitting scheme, the overall amount of data used for training the model is 980 for each class. The remaining 240 from each class were used for validation. After successfully completing the data distribution using splitting scheme, the AlexNet and ResNet 50 models were trained until the networks attain the best performance. Finally, the model with the best validation accuracy is saved and tested with the testing dataset, which is 120 for each class.

**Fig. 8:**
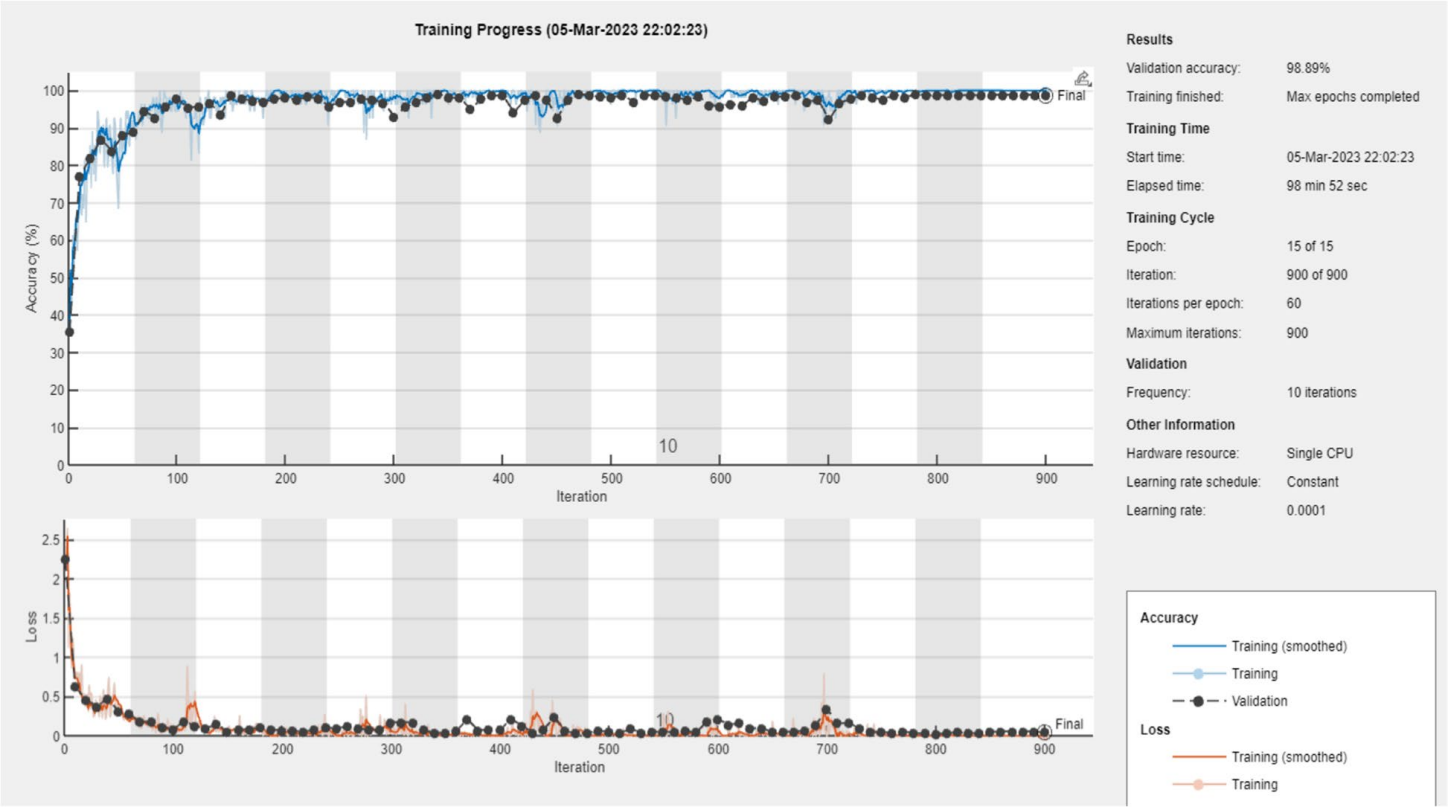
Training and validation accuracy curve of AlexNet

**Fig. 9:**
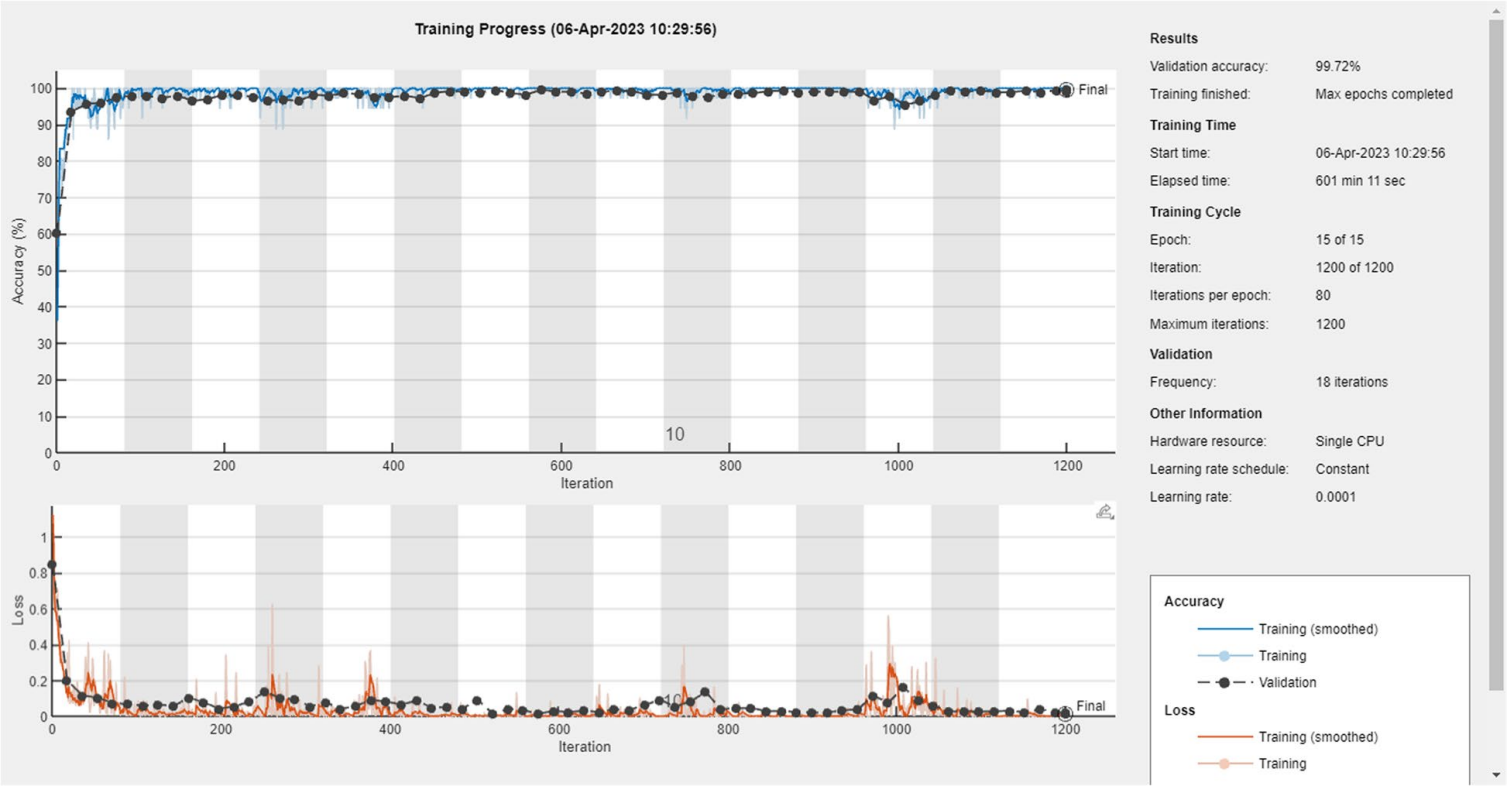
Training and validation accuracy curve of ResNet50

The curve plot shown in Figs. 8 and 9 describes the training and validation performance of AlexNet and ResNet 50 neural networks respectively. In the curve plot, the learning curve is plotted from the training performance of the model on the training data set and this plot shows the learning capability of the models. Capability of the model’s generalization is plotted on validation curve which is derived from validation dataset. Moreover, validation and training loss is also represented in the curve plot to show the converging ability of the loss.

**Fig. 10:**
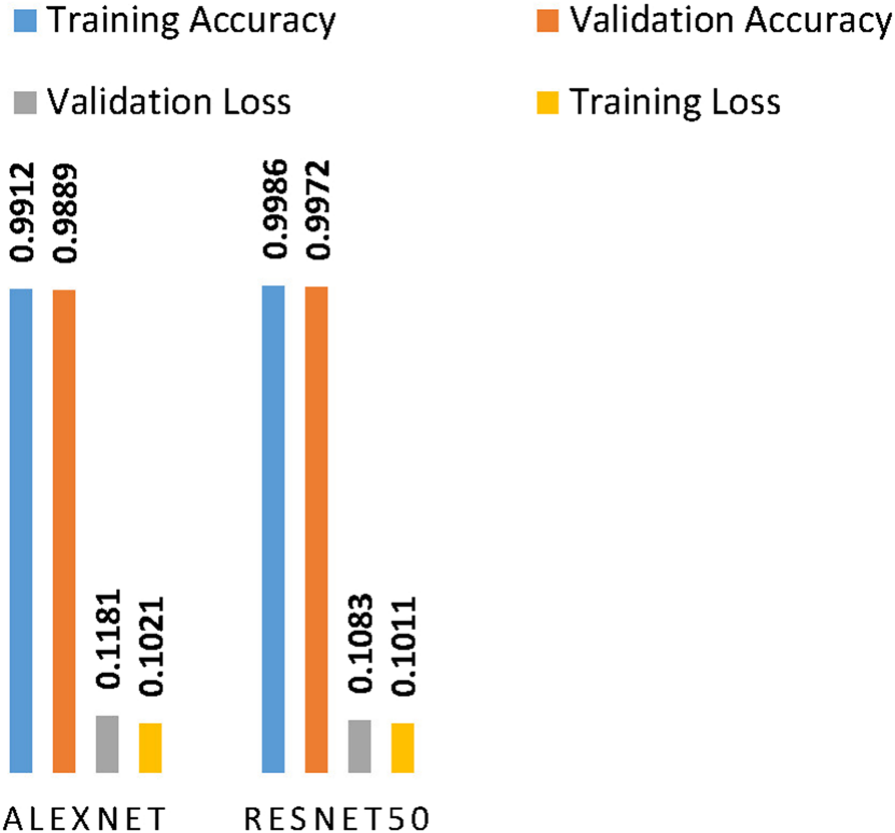
Training performance summary of AlexNet and ResNet 50 models

During training, the lowest training loss achieved was 0.1021, and the validation loss was 0.118 for AlexNet. For the ResNet 50 model, a training loss of 0.1011 and a validation loss of 0.1080 were achieved. When we examine the accuracy of validation and training from the curve plot of Fig. 8, the AlexNet model achieved 99.1% and 98. 89, respectively. Examining the ResNet 50 model curve from Fig. 9, it can be noted that the model achieved training and validation accuracies of 99.86% and 99.72%, respectively. For more clarification of the training and validation curve results, the performance of both models is summarized in Fig. 10.

### Testing result

When examining the validation accuracy and validation loss results of the proposed models, the ResNet50 model showed superior performance over AlexNet in classifying ECG signals. As is clearly seen in the curve plot and summary of the result, the ResNet-50 model achieved better performance of learning accuracy, validation accuracy and convergence in both validation and training. Although the performance of the AlexNet model was good, signs of overfitting were observed during training in several epochs, which may affect the robustness of the model in classifying unseen data. Therefore, based on the training and validation results, ResNet-50 was selected to perform the classification task on the unseen testing data. In testing phase varies performance evaluation metrics such as: precision, recall, sensitivity, specificity and accuracy were used. Thus, the ResNet 50 has expected to classify given input test data in to ARR, CHF and NSR class. Thus, in testing performance of the model was evaluated for different performance metrics such as classification accuracy, sensitivity and specificity. In classification, the model is expected to categorize given input data into ARR, CHF and NSR classes.

**Fig. 11:**
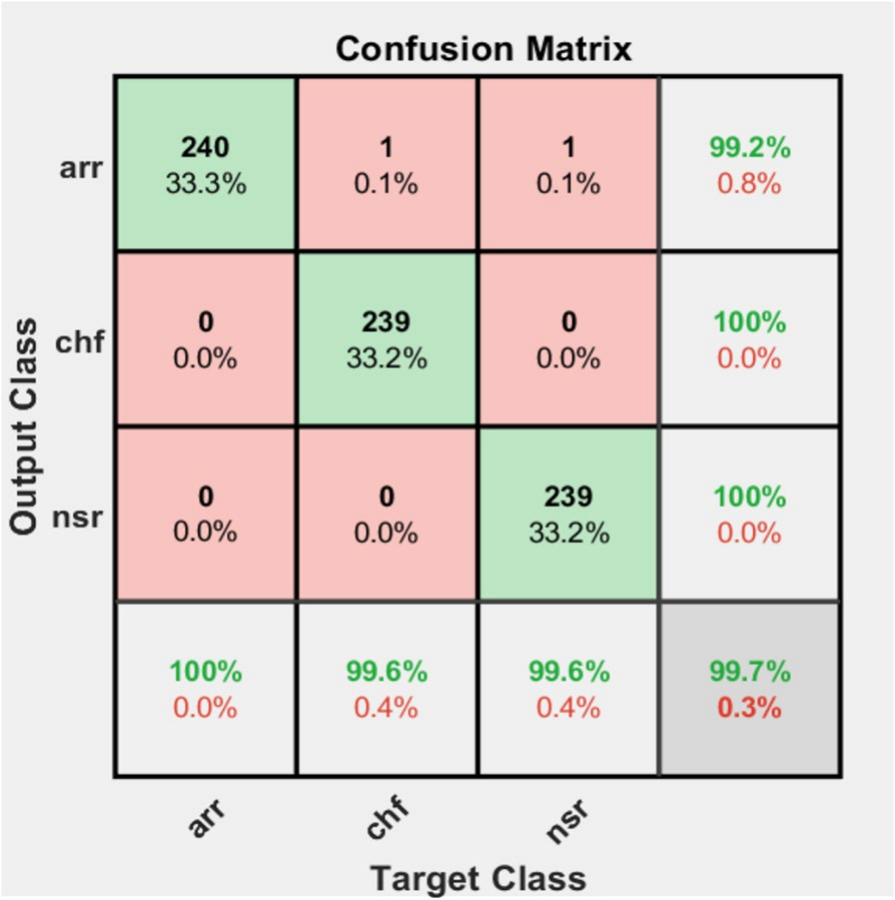
Confusion matrix result of test data

The confusion matrix shown in Fig. 11 is exported from MATLAB. From the figure it can be noted that the rows represent the predicted class by our model (also called output class), whereas the columns indicate to the actual class of the test data (also called target class). Both the percentage and the number of observations is shown in each cell. The diagonal cell coloured with green represents the number of data correctly classified in the corresponding class. The reddish coloured cells that are off the diagonal cell represents the number of data that are wrongly classified in the corresponding class. The white cells of the last column of the confusion matrix plot correspond to the percentages of prediction that the test data to belong to a class that is correctly identified (green text) and incorrectly (red text) classified. This result represented in green text is called precision or models capability to predict positive result. The white cells of last row of the confusion matrix represent the percentages of all the test data belonging to each class of the ECG that are correctly (green text) and incorrect (red text) classified. These values are often called the recall or prediction ability of the true positive result. Furthermore, the gray cell in the last row of the confusion matrix plot shows the overall accuracy of the ResNet 50 in classification of test data.

According to the confusion matrix, all 120 ARR data are classified correctly, and two of the 120 CHF classes are misclassified as ARR and NSR. From 120 testing data of NSR, only one is misclassified as ARR. From Table 1, the true positive, false positive, true negative and false negative classification values are easily known. Hence, varies performance evaluation metrics can be calculated using these values. Overall classification accuracies of the model are represented at the last cell of the fourth column indicated by grey cell. The first three cells of fourth column represent recall value of the model for each of the corresponding classes and the first three cells of last row represent precision of the model for each of the corresponding classes. So, from the confusion matrix the performance evaluation of the model is summarized as shown in Table 1.

**Table 1:** Summary of performance matrices on test data

Class	TP	TN	FP	FN	Se	Sp	Pr	Re	F1
ARR	120	238	2	0	100%	99.2%	98.4%	100%	99.2%
CHF	118	240	0	2	98.3%	100%	100%	98.3%	99.1%
NSR	119	239	1	1	99.2%	99.6%	99.2%	99.2%	99.2%
					Avg = 99.2%	Avg = 99.6%	Avg = 99.2%	Avg = 99.2%	Avg = 99.2%

As described in Table 1 above, accuracy of its 99.2%, average sensitivity of 99.2%, average specificity of 99.2%, average F1 score of 99.2% is achieved on classification of ECG signals using the ResNet-50 transfer learning approach.

## Discussion

The electrocardiogram is the gold standard diagnostic and monitoring tool for CVD in modern medicine. It is a tool that accounts for the electrical activity of the heart recorded by using a skin electrode noninvasively over the chest. The overall diagnosis result of ECG is depicted in waveform pattern and shape enabling doctors to identify diseases.

Since ECG signals are non-stationary signal, they are dynamic in nature. Hence, anomalies may not occur at all times of recording. Therefore, doctors require long hours for observation and assessment of the waveform to diagnose anomalies correctly. However, examining of ECG for a long hour requires large amounts of data and this turns out to be tiresome and time consuming. Moreover, due to the amount of data used in analysis, the probability of missing data is very high. Therefore, an automated system that distinguishes abnormal and normal ECG signals is required to assist doctors for easy identification of cardiac arrhythmia. To achieve this, pretrained deep learning models AlexNet and ResNet 50 were proposed in this study and implemented on MATLAB 2022 a. Before training both models, the time series 1D ECG signals were preprocessed and converted into a 2D image using Morse wavelet. Then, the pertained ResNet 50 and AlexNet models were fine-tuned on ECG data. During training, the validation and training performance of both models were compared to select the best model for the classification task of the test data; thus, the ResNet 50 model was selected for classification. To achieve this learning and classification task, the models were evaluated using two metrics: accuracy and loss metrics. From the accuracy and loss metrics of the training and validation curve plotted in Figs. 7 and 8, the AlexNet and ResNet 50 performances were evaluated and compared.

Doing so, the ResNet network model optimized with Adam, fine-tuned with learning rate of 0.0001, minibatch size of 20 and frequency of validation of 18 achieved best training and validation accuracy at the 15^th^ epoch in comparison with AlexNet optimized and fine-tuned with similar optimizer and hyper parameters. Thus, validation accuracies of 99.72% and 98.89% were achieved for the AlexNet and ResNet 50 models, respectively. Moreover, the lowest training loss achieved was 0.1021, and the validation loss was 0.118 for AlexNet. A training loss of 0.1011 and a validation loss of 0.1080 were achieved for ResNet 50.

After comparing the training results, the ResNet 50 model network with the best training and validation performance was saved and finally tested with 120 testing data points preserved for each class of ECG data. As illustrated in the Fig. 11 confusion matrix, the promising classification performance was achieved on the testing data with overall accuracy of 99.2%, average sensitivity of 99.2%, average specificity of 99.6%, average recall, precision and F- measure of 99.2%.

The proposed approach showed excellent classification results for all classes and outperformed the other methods in the detection of arrhythmia. Thus, the result achieved in the present state of the art is comparable with most related works of deep learning experimented on the same database. In [12], a pretrained EfficientNet B0 convolution neural network model was implemented. However, STFT is used for converting 1D ECG signals into 2D images, thus achieving a classification accuracy of 97.3% when testing the model using the PhysioNet dataset. A hybrid deep learning model was used in [13] to classify the PhysioNet MIT-BIH arrhythmia database based on CNN and BLSTM approaches for arrhythmia identification and achieved a classification accuracy of 97.15%. Karthiga et al. proposed a deep learning convolutional neural network for the classification of ECG signals from the MIT-BIH Arrhythmia database, and 91.92% classification accuracy was reported [14]. A hybrid deep learning model named CNN—LSTM was proposed in [15] for cardiac arrhythmia detection from CWT images of ECG recordings. According to reported result, the proposed approach provided 98.0%, 96.0%, and 98.0% accuracy for ARR, CHF, and NSR, respectively. Compared with the aforementioned literature, which implemented various deep learning models, on the same database, our model achieved a better classification task for classifying the ECG signal into three classes: ARR, CHF and NSR. A summary of the most related works conducted on the same database is reported in Table 2 below.

**Table 2:** Summary of the most related works and current work

Author	Database	Data Input	Approach	Performance		
				**Acc**	**Sen**	**Sp**
Alfaras et al. [6]	MIT-BIH arrhythmia database	1 D ECG	Ensemble of Echo State Networks	96.8%	92.7%	-
Kishore et al. [8]	MIT-BIH arrhythmia database	1 D ECG	Radial Basis Function Neural Network	98.5%	98.3%	99%
Ting et al. [12]	MIT-BIH arrhythmia database	2D ECG	EfficientNet B0	97.3%	89.55%	89.55%
Bhatia et al. [13]	MIT-BIH arrhythmia database	1D ECG	LSTM + CNN	98.36%	-	94.36%
Karthiga et al. [14]	MIT-BIH arrhythmia database	2D ECG	CNN	91.92%	90.21%	95.18%
Madan et al. [15]	MIT-BIH arrhythmia database	2D ECG	LSTM + CNN	97.3%	97%	98%
**Proposed work**	MIT-BIH arrhythmia database	2D ECG	ResNet 50	99.2%	99.2%	99.6%

## Conclusion

In present work two distinct works have been done. First the 1D ECG signal is preprocessed and converted to 2D images using Morse wavelets. This method of representing the time series signal in image form is very effective in revealing the hidden and visible characteristics of nonstationary signals in the time and frequency domains simultaneously. Due to this characteristic, it is considered as an ideal choice for converting a 1D ECG into an image. Thus, Morse wavelet with γ value of 3, P2 value of 60, sampling frequency of 128 Hz and voice for an octave value of 12 was used in MATLAB for conversion of 1D ECG signal to image in this study. Secondly, pretrained AlexNet and ResNet 50 were fine-tuned with hyperparameters and optimized with optimizer for ECG data classification. Therefore, ResNet50 has shown the best training and validation performance over AlexNet. Despite the fact that the ResNet 50 model has shown remarkable training and validation performance over AlexNet mode, the computation time of ResNet during training was very high in comparison with AlexNet due to the depth of the network. Hence, it is recommended to use GPUs for training such a deep neural network.

Moreover, the proposed method only focused on classifying the ECG signal into three classes, so it is recommended to increase the class of the ECG data for future work by collecting more data. Although the approach is efficient in classifying arrhythmia, normal sinus rhythm and congestive heart failure using the PhysioNet database, its efficacy must be evaluated with real-time data.

## Data Availability

The datasets and materials used in the current study are available publicly, Links to the datasets is provided in the data source section.

## Abbreviations

ARR: Arrhythmias
CHF: Congestive Heart Failure
CNN: Convolutional Neural Network
CVD: Cardiovascular Disorder
CWT: Continuous Wavelet Transform
ECG: Electrocardiogram
EEG: Electroencephalogram
EMG: Electromyogram
FHR: Fatal Heart Rate
LRN: Local Response Normalization
LSTM: Long Short Term memory
ResNet: Residual Network
ReLU: Rectified Linear Unit
STFT: Short Time Fourier Transform
WHO: World Health Organization
